# Automatically Recognizing Medication and Adverse Event Information From Food and Drug Administration’s Adverse Event Reporting System Narratives

**DOI:** 10.2196/medinform.3022

**Published:** 2014-06-27

**Authors:** Balaji Polepalli Ramesh, Steven M Belknap, Zuofeng Li, Nadya Frid, Dennis P West, Hong Yu

**Affiliations:** ^1^University of Massachusetts Medical SchoolWorcester, MAUnited States; ^2^University of Wisconsin MilwaukeeMilwaukee, WIUnited States; ^3^Northwestern University Feinberg School of MedicineDepartment of Dermatology and Department of Medicine, Division of General Internal Medicine and GeriatricsChicago, ILUnited States; ^4^Northwestern University Feinberg School of MedicineDepartment of DermatologyChicago, ILUnited States; ^5^VA Central Western MassachusettsLeeds, MAUnited States; ^6^University of MassachusettsDepartment of Computer ScienceAmherst, MAUnited States

**Keywords:** natural language processing, pharmacovigilance, adverse drug events

## Abstract

**Background:**

The Food and Drug Administration’s (FDA) Adverse Event Reporting System (FAERS) is a repository of spontaneously-reported adverse drug events (ADEs) for FDA-approved prescription drugs. FAERS reports include both structured reports and unstructured narratives. The narratives often include essential information for evaluation of the severity, causality, and description of ADEs that are not present in the structured data. The timely identification of unknown toxicities of prescription drugs is an important, unsolved problem.

**Objective:**

The objective of this study was to develop an annotated corpus of FAERS narratives and biomedical named entity tagger to automatically identify ADE related information in the FAERS narratives.

**Methods:**

We developed an annotation guideline and annotate medication information and adverse event related entities on 122 FAERS narratives comprising approximately 23,000 word tokens. A named entity tagger using supervised machine learning approaches was built for detecting medication information and adverse event entities using various categories of features.

**Results:**

The annotated corpus had an agreement of over .9 Cohen’s kappa for medication and adverse event entities. The best performing tagger achieves an overall performance of 0.73 F1 score for detection of medication, adverse event and other named entities.

**Conclusions:**

In this study, we developed an annotated corpus of FAERS narratives and machine learning based models for automatically extracting medication and adverse event information from the FAERS narratives. Our study is an important step towards enriching the FAERS data for postmarketing pharmacovigilance.

##  Introduction

### Background

**Figure 1:**
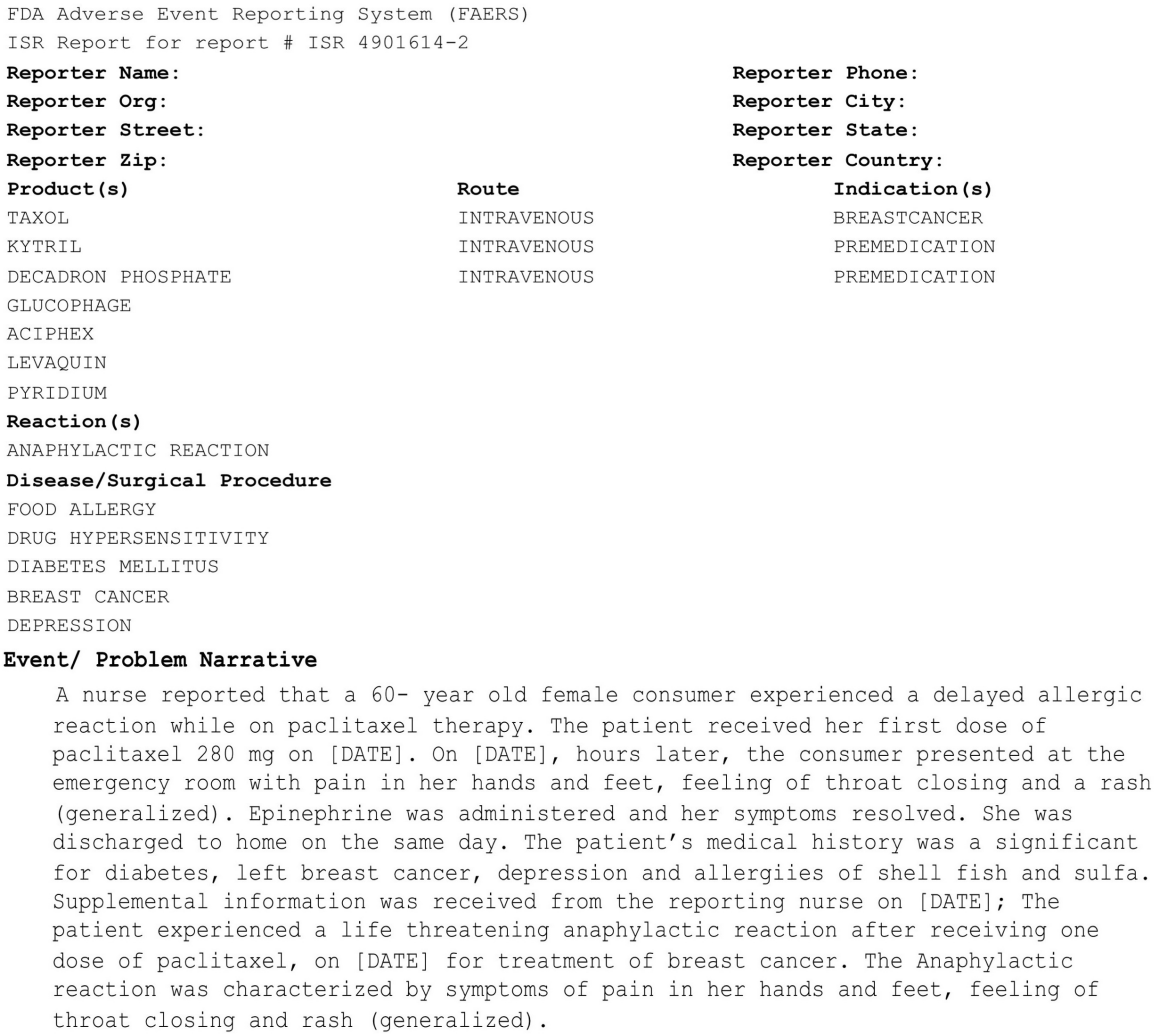
A sample AERS Report with structured data and narrative text.

An adverse event (AE) is an injury or untoward medical occurrence to a patient or clinical investigation subject who has been administered a pharmaceutical product and the AE does not necessarily have a causal relationship with the administered treatment [1,2]. An adverse drug event (ADE) is an injury resulting from a medical intervention related to a drug, including harm caused by the drug (adverse drug reactions and overdoses), and harm from the use of the drug (including dose reductions and discontinuations of drug therapy) [3,4]. Studies have reported that ADEs account for nearly 20% of all adverse events that occur in hospitalized patients [5-7]. In the United States alone, ADEs account for more than 770,000 injuries and deaths annually [8-10], and an increased average length of stay in hospitals at a cost of between $1.56 and $5.60 billion annually [3,11]. Improved methods for ADE detection and analysis may identify novel drug safety signals and lead to improved methods for avoiding ADEs, with their attendant burden of morbidity, mortality, and cost. As part of a major effort to support postmarketing drug safety surveillance, the US Food and Drug Administration (FDA) receives mandatory reports on ADEs from manufacturers through the FDA Adverse Event Reporting System (FAERS). The FAERS is a database that captures information concerning adverse events and medication errors associated with FDA-approved prescription drugs. Currently, FAERS contains over four million reports of adverse events dating from 1969 to present [12]. It serves as a rich resource for pharmacovigilance-the study of drug-related injuries for the purpose of making warning or withdrawal recommendations for pharmaceutical products [4]. A typical FAERS report incorporates both structured data and unstructured free text, as shown in Figure 1. The structured data entries incorporate each patient’s personal and demographic information, a list of prescribed drugs, and the class of drug reaction (in this example, “anaphylactic reaction”) (Figure 1). The Event/Problem narrative contains additional information relevant to describing the event, assessing causality, and grading severity (Figure 1). In this example, the narrative text contains the phrase that indicates causality between paclitaxel and the anaphylactic reaction while “experienced a life threatening anaphylactic reaction” shows the severity of the event, which is not coded in the structured data.

Although FAERS is an excellent resource to study drug effects, as stated in Tatonetti et al [13], the structured data does not incorporate confounding factors including concomitant medications and patient medical histories, which limits FAERS’ effectiveness for pharmacovigilance. In contrast, such confounding factors are frequently described in the FAERS narratives. Making this data computationally available is critical for pharmacovigilance.

Currently, manual abstraction is required for identification of relevant data in FAERS narratives. Manual abstraction is expensive and often not practical, given the current size of the FAERS dataset, which contains millions of records. Therefore, it is important to develop computational approaches to automatically extract information from FAERS narratives. In this study, we report the development of both a corpus of FAERS narratives annotated with medication and adverse event information and a Natural Language Processing (NLP) system called AETagger that automatically extracts this information from the narratives and is adapted from existing tools. This is an important step towards enriching the existing FAERS’ capacity for pharmacovigilance.

### Related Work

There is extensive research related to AE and ADE detection and analysis from a variety of data sources. Earlier work examined patients’ paper medical records determining whether AEs and ADEs can be reliably abstracted based on the information conveyed in those records. For example, Hiatt et al (1989) [14] was among one of the early studies that defined an AE as an injury caused at least in part by medical mismanagement (negligence). They then manually abstracted ADEs from patients’ paper-based clinical medical records. Similarly, other early studies (eg, [3,7,15]) defined AEs and ADEs and manually abstracted them from clinical records. These studies indicate the feasibility and value of clinical records for ADE surveillance and prevention.

When electronic medical records (EMRs) became available, computational approaches were developed to automatically identify AE and ADE information from EMRs. Studies used rule-based approaches for detecting ADEs from EMR data [16-18]. Tinoco et al [19] compared a rule-based computer surveillance system called Health Evaluation through Logical Processing (HELP) [20] with manual chart reviews on 2137 patient admissions. They reported that HELP detected as many ADEs as were found by manual chart review, suggesting that NLP systems could improve ADE detection from EMR narrative data.

Many studies applied NLP to detect AEs and then inferred a causality relationship between a drug and an AE (called an ADE) using logical rules, statistical analyses, and supervised machine learning (ML) approaches. Hazlehurst et al [21] developed MediClass, a knowledge-based system that deploys a set of domain-specific logical rules to medical concepts that are automatically identified from EMR narratives (eg, progress notes) or precoded data elements (eg, medication orders). The system achieved a precision of 64% for detecting vaccine-related AEs [22]. A number of studies applied the NLP system [23-25], MedLEE [26], to detect AEs from discharge summaries and hospitalization records. For example, Wang et al [23] applied MedLEE to detect terms and mapped them to the Unified Medical Language System (UMLS) semantic types. Subsequently, they detected medication and AEs when the terms were mapped to the UMLS concepts with the semantic types of Clinical Drug (T200) and Disease or Symptom (T047), respectively. The causality relationship between a medication and an AE was extracted from 25K discharge summaries based on a χ^2^-statistical analysis of medication and AE. Evaluation of seven drugs for known ADEs led to a recall and precision of 75% and 31% respectively. Aramaki et al [27] manually annotated 435 discharge summaries for drugs and ADEs and then applied supervised machine learning techniques to detect these named entities. They identified the causality between drugs and AEs using pattern matching and SVM techniques. They reported a recall and precision score of 0.81 and 0.87 for drug, and 0.80 and 0.86 for AE detection respectively. For inferring causality they achieved recall and precision of 0.92 and 0.41 using pattern matching, and 0.62 and 0.58 using SVM technique respectively.

In addition to EMRs, studies have explored other data sources for ADE information, including biomedical literature [28,29], social media and the Internet [30-32]. Shetty and Dalal [33] mined ADEs from PubMed citations. They first built a document classifier to identify relevant documents that incorporate ADE relationships using Medical Subject Headings (MeSH) terms. For example, if an article is assigned “chemically induced” or “adverse effects,” then the article is likely to incorporate an ADE. They then identified ADE signals using disproportionality analysis in which the rate at which a particular AE of interest co-occurs with a given drug is compared to the rate an AE occurs without the drug in the collection. Their evaluation on a predefined set of 38 drugs and 55 AEs showed that their literature-based approach could uncover 54% of ADEs prior to FDA warnings.

There is a rich store of literature for ADE detection on Spontaneous Reporting Systems (SRS) such as the FAERS reports and WHO VigiBase [34]. Studies have explored several statistical data mining and machine learning techniques on SRS for the detection of ADE signals [13,35-60]. However, all aforementioned approaches for ADE detection from FAERS are based on its structured data. In this study, we report the development and evaluation of supervised machine learning approaches for automatically detecting medication information and adverse events from the FAERS narratives. We speculate that such information can be a useful addition to the FAERS structured data for ADE detection.

## Methods

### Annotation Data and Procedure

Through our collaboration at Northwestern University [61], we obtained a collection of 150 de-identified FAERS narratives; a sample is shown in Figure 1. The data collection originally came as a scanned PDF image file. With Institutional Review Board (IRB) approval from Northwestern University and University of Wisconsin Milwaukee, we manually transcribed the PDF file into a computer-readable text file.

We randomly selected a set of 28 narratives for developing the annotation guideline (Multimedia Appendix 1). Our annotation guideline was based on the i2b2 challenges in NLP for Clinical Data Medication Extraction [62,63]. A balanced interdisciplinary team consisting of a linguist (NF), a physician (SB), two informaticians (BPR and HY) and a physician informatician (ZFL) developed the annotation guideline through an iterative process. At the end of reviewing 28 narratives, we obtained a guideline that all the members of the team agreed upon.

Following the final annotation guideline, two annotators (ZFL, designated as AnnPhy, and NF, designated as AnnLing), both of whom were the primary annotators for the i2b2 medication event detection challenge [63] in which we participated, independently annotated the remaining 122 AERS narratives. The different backgrounds of the annotators aids in building a corpus that is both linguistically driven and clinically correct. A physician (SB) served as a tiebreaker and resolved annotation disagreements. This collection of 122 narratives is comprised of approximately 23,000 word tokens and the average number of words per narrative is 190.2 (SD 130.3).

The annotation was carried out using Knowtator [64], a plugin for Protégé [65]. The Knowtator interface allows users to define entities that need to be annotated and configure the relationships between them. The 122 annotated narratives were used as both training and testing data for machine learning approaches described below. The annotated data was grouped into four collections each containing 122 narratives: *AnnPhy* and *AnnLing* –data annotated by annotators AnnPhy (ZFL) and AnnLing (NF), respectively; *Comb* –a joint set of annotations agreed upon by both AnnPhy and AnnLing, and *Tie –*a joint set of AnnPhy and AnnLing annotations where disagreements were resolved by the tiebreaker SB. We also report Cohen’s kappa, a well-known statistic used to assess the degree of Inter-Annotator Agreement (IAA) between annotators [66]. We use these four sets of data to capture all named entities and build robust supervised machine learning classifiers to identify them.

### Supervised Machine Learning

#### Machine Learning Techniques

Three supervised machine learning approaches were explored for automatically identifying medication information and adverse events: Naïve Bayes (NB), Support Vector Machines (SVMs) and Conditional Random Fields (CRFs) [67]. We built NB and SVM classifiers using Weka [68] and the CRF model was built using the A Biomedical Named Entity Recognizer (ABNER) toolkit [69]. NB is a simple model that assumes all attributes of the examples which are independent of each other given the context of the class. SVMs are a well-known statistical machine learning algorithm and have shown very good performance in many classification tasks [70,71]. CRFs have shown success in named entity recognition in the biomedical domain [69,72].

#### Learning Features

We explored a variety of features such as syntactic features, semantic features based on the external knowledge resource (UMLS), morphological and contextual features, presence of negation, hedging and discourse connectives as a feature in addition to ABNER default features which include bag of words and orthogonal features. We describe each of these in detail below.

**Figure 2:**
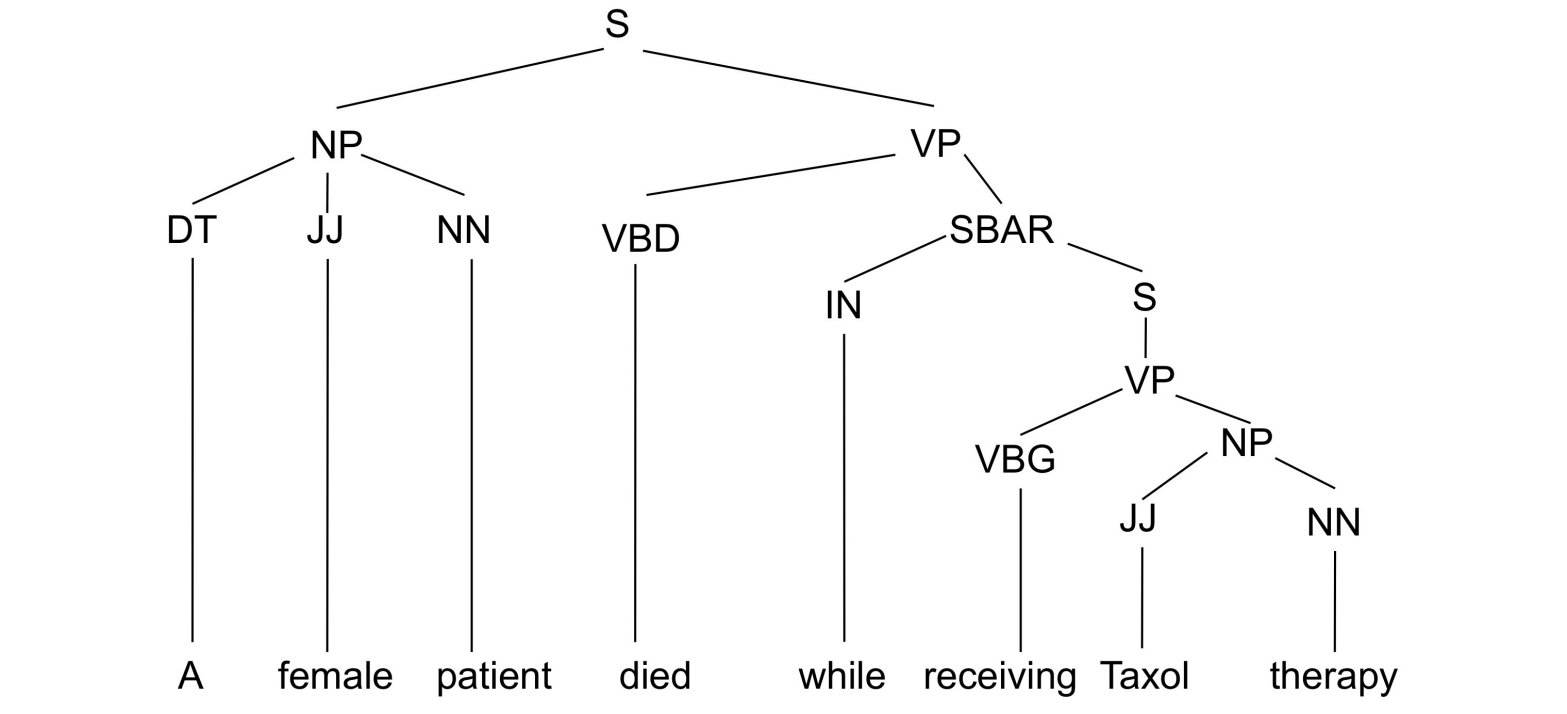
The sample constituency parse tree. S: simple declarative clause, NP: noun phrase, VP: verb phrase, DT: determiner, JJ: adjective, NN: noun, VBD: verb, past tense, SBAR: subordinate clause, IN: preposition or subordinating conjunction, VBG: verb, gerund or present participle.

The syntactic features include the part-of-speech (POS), the phrasal class of each token, and the POS of the token immediately to the left of the token under consideration. The syntactic features were extracted from the constituency parse tree generated by the Charniak-Johnson parser [73] trained in the biomedical domain. This parser was determined to have the best performance when tested on the GENIA corpus [74]. Figure 2 shows a sample constituency parse tree. In this example, the POS features determiner (DT), adjective (JJ), noun (NN) are the POS of tokens “A”, “female”, and “patient” respectively. Further, the phrasal class for all the three tokens is noun phrase (NP). The left sibling POS value of “A” is NONE assuming it is the start of the sentence. The left sibling POS of “female” and “patient” tokens are DT and JJ respectively.

We applied the UMLS Metamap [75,76] to extract semantic features, which are concepts and semantic types represented in the UMLS Metathesaurus. The morphological features were obtained by considering various characteristics of the word. We took attributes of the word, such as whether it was a digit, was capitalized, its alphanumeric order (ie, if the token started with letters and was followed by numerals or vice versa), and the presence of punctuation such as commas and hyphens. These features were extracted using a simple pattern-matching technique. The first (prefix) and last (suffix) three and four characters of the token were added as affix features.

We added as features, negation and hedging cues with their scope that were detected automatically by the systems described in the literature [77,78]. We also added presence of discourse connectives that were automatically detected by the discourse parser [79].

### Systems

#### Overview

We developed several taggers to evaluate the complexity of the task for identifying medication information and adverse events and the impact of features.

#### Systems to Evaluate Task Complexity

In this experiment, we built two baseline systems to compare the performance of ML algorithms. The first system *BaseDict* is a simple dictionary-matching system. A lexicon of medications and AEs is compiled from the UMLS Metathesaurus using the semantic types as defined by Wang et al [23], where terms having the semantic types Clinical Drug (T200) and Disease or Symptom (T047) were considered as drug and adverse event respectively. The baseline system *BaseDict*, tags all instances of the lexicon that match within the text. The second system, *MetaMapTagger*, is a UMLS Metamap [75] based system that tags phrases as AEs or medications using UMLS semantic types similar to *BaseDict*.

The baseline systems were compared with taggers built using bag of words as the default feature –*NBTagger*, a NB-based tagger, *SVMTagger*, a SVM-based tagger, and *SimpleTagger*, a CRF-based tagger built using ABNER default features. We then evaluate the taggers by adding all the features defined in the Learning Features section, which we call *NBTagger* ^*+*^, *SVMTagger* ^*+*^ and *CombinedTagger* for NB, SVM- and CRF-based taggers respectively.

#### Systems to Evaluate Impact of Features

We evaluate the impact of various features on the performance of tagger. We used the ML technique found to have the best performance in our previous experiment. In addition to the default features trained as *SimpleTagger*, we individually added syntactic features (*SyntacticTagger),* semantic features (*SemanticTagger*), morphological features (*MorphologicalTagger*), affix features (*AffixTagger)*, negation and hedging features (*NegHedgeTagger*), discourse connective features (*ConnectiveTagger*), and a tagger incorporating all the features (*CombinedTagger*) which were trained to identify the named entities.

### Machine Learning Evaluation Metrics

All the AE taggers trained were evaluated using ten-fold cross-validation. We reported recall, precision, and F1 score. Recall is the ratio of the number of entities of a certain class correctly identified by the system and the number of entities of that class in the gold standard. Precision is the ratio of the number of entities of a certain class correctly identified by the system and the number of entities of that class predicted by the system. F1 score is the harmonic mean of precision and recall.

## Results

### Corpus Characteristics and Annotation Agreement

Table 1 shows the definitions of adverse event and medication-related named entities, the number of annotated instances, and Cohen’s kappa value. The annotation agreement is calculated based on two criteria: *strict* in which the two annotations have an exact match, and *unstrict* in which there exists an overlap of at least one word between the two annotations. We measured the agreement using *unstrict* criteria to estimate the agreement between annotators when entity boundary is ignored. The table also shows the number of instances annotated in all four data sets.

As shown in Table 1, *adverse event* (*AE*) was the most frequently annotated entity followed by *medication* entity. *Duration* had the least number of annotated instances and lowest kappa value (.34) for *strict* criteria. *Indication* had the second highest kappa value for *unstrict* criteria (.93) after *medication* (.95), since most of the *indication* entities were followed by explicit and unambiguous patterns such as “for the treatment of”, “diagnosed with”, “due to”, “enrolled in breast cancer study”, and so on.

**Table 1:** Named entity definition, number of annotated instances, and inter-annotator agreement measured by Cohen’s kappa for both strict and unstrict criterion.

Named entity	Definition	Number of instances annotated					kappa (strict)	kappa (unstrict)
		AnnPhy		AnnLing	Comb	Tie		
Medication	Name of the drug they administered to patient including drug class name or medications referred to with	1231		1278	1152	1286	.92	.95
Dosage	Amount of a single medication used in each administration	143		315	137	205	.59	.82
Route	Method for administering the medication	115		244	107	132	.59	.64
Frequency	How often each dose of the medication should be taken	25		56	21	42	.58	.74
Duration	How long the medication is to be administered	34		153	24	51	.34	.87
Indication	Medical conditions for which the medication is given	175		148	126	175	.76	.93
Adverse event (AE)	Harm directly caused including the pronouns referring to it by the drug at normal doses and during normal use	1689		2083	1646	1842	.83	.93
Other signs, symptoms and diseases (OSSD)	Other symptoms associated with the disease	234		140	90	147	.50	.71
Treatment	Treatment the patient received for the disease	77		216	62	153	.39	.77
Total		3723		4633	3365	4033		

### Results of Supervised Learning

Table 2 reports recall, precision, and F1 score of the AETaggers for identifying the AE and other medication-related named entities on each of the four data sets as described in Annotation and data procedure section.

The baseline system *BaseDict* that matches only *AE* and *medication* achieved an F1 score of 0.45, 0.41, 0.46, and 0.42 on the *AnnPhy*, *AnnLing*, *Comb*, and *Tie* datasets respectively. The *MetamapTagger* also had similar performance. Among the taggers using bag of words as features, the CRF-based *SimpleTagger* had the best performance. The addition of features improved the performance of the ML classifiers. The *CombinedTagger* achieved best performance with F1 scores of 0.69, 0.74, and 0.73 on the *AnnPhy*, *AnnLing*, and *Comb* datasets respectively. The *SVMTagger* ^*+*^ had the best performance with a 0.66 F1 score on the *Tie* dataset. The difference in performance between *CombinedTagger* and *SVMTagger* ^*+*^ taggers was statistically significant only on *AnnLing* dataset (*t* test, *P*=.003). The ML-based taggers clearly outperform the baseline method. The CRF-based tagger had the best overall performance and was therefore chosen as the system to be adopted for subsequent experiments measuring impact of features.

We trained the CRF-based AETaggers using different features as described in the Learning Features section. The results show that the *CombinedTagger* achieved the highest performance on all datasets. Our results also show that the *AnnLing* dataset has the highest performance while *Tie* performs the lowest. *Comb* outperforms both *Tie* and *AnnPhy*.

Since the *Comb* dataset’s performance (0.73 F1 score) is close to the highest (0.74 F1 score) and contains annotations agreed upon by both annotators, we further report feature analyses using the *Comb* dataset. Table 3 shows how different learning features affect AETagger’s performance. The results show that adding a single feature added little to the overall performance, although the performance of different entities varied. Affix features improved *route* and *duration* but decreased *AE*, *medication,* and *dosage*. Connective features increased the performance of *dosage*, *route*, and *indication*; however, the performance of *medication* decreased. Other features (morphological, negation, hedge, semantic, and syntactic) showed similar patterns. On the other hand, when all features were added, the overall performance increased to 0.73 F1 score (default 0.71), although the increase was not statistically significant (*t* test, *P*=.08).

**Table 2:** The precision, recall, and F1 score of Taggers on each of the four annotated data sets (*t* test, *P*<.01).

Machine learning		AnnPhy Mean (SD)				AnnLing Mean (SD)			Combined Mean (SD)				Tie Mean (SD)			
		F1		Precision	Recall	F1	Precision	Recall	F1		Precision	Recall	F1		Precision	Recall
**Task complexity**																
	BaseDict	0.45 (0.10)		0.86 (0.08)	0.31 (0.09)	0.41 (0.09)	0.91 (0.07)	0.27 (0.08)	0.46 (0.12)		0.82 (0.06)	0.32 (0.11)	0.42 (0.10)		0.86 (0.13)	0.28 (0.08)
	MetaMapTagger	0.41 (0.17)		0.41 (0.16)	0.42 (0.18)	0.41 (0.10)	0.47 (0.20)	0.37 (0.15)	0.42 (0.18)		0.41 (0.17)	0.43 (0.19)	0.40 (0.16)		0.46 (0.19)	0.36 (0.14)
	NBTagger	0.22 (0.08)		0.39 (0.17)	0.15 (0.05)	0.23 (0.08)	0.45 (0.14)	0.16 (0.06)	0.24 (0.08)		0.40 (0.17)	0.17 (0.05)	0.20 (0.06)		0.47 (0.19)	0.13 (0.04)
	SVMTagger	0.55 (0.05)		0.77 (0.10)	0.44 (0.04)	0.55 (0.05)	0.78 (0.07)	0.43 (0.05)	0.58 (0.04)		0.78 (0.10)	0.46 (0.04)	0.59 (0.04)		0.80 (0.05)	0.46 (0.05)
	SimpleTagger	0.67 (0.09)		0.77 (0.09)	0.60 (0.09)	0.72 (0.08)	0.81 (0.06)	0.66 (0.10)	0.71 (0.08)		0.81 (0.09)	0.63 (0.08)	0.63 (0.09)		0.69 (0.08)	0.55 (0.10)
	NBTagger^+^	0.45 (0.09)		0.38 (0.10)	0.56 (0.06)	0.44 (0.06)	0.39 (0.07)	0.50 (0.06)	0.46 (0.09)		0.37 (0.11)	0.60 (0.04)	0.43 (0.07)		0.38 (0.08)	0.51 (0.07)
	SVMTagger^+^	0.66 (0.07)		0.78 (0.10)	0.58 (0.06)	0.67 (0.07)	0.78 (0.07)	0.59 (0.07)	0.70 (0.06)		0.80 (0.11)	0.63 (0.05)	0.66 (0.07)		0.78 (0.06)	0.57 (0.08)
	CombinedTagger	0.69 (0.09)		0.77 (0.10)	0.62 (0.09)	0.74 (0.08)*	0.81 (0.07)	0.68 (0.09)	0.73 (0.08)		0.81 (0.10)	0.66 (0.07)	0.65 (0.08)		0.71 (0.08)	0.60 (0.09)
**Impact of features**																
	SimpleTagger	0.67 (0.09)		0.77 (0.09)	0.60 (0.09)	0.72 (0.08)	0.81 (0.06)	0.66 (0.10)	0.71 (0.08)		0.81 (0.09)	0.63 (0.08)	0.63 (0.09)		0.69 (0.08)	0.55 (0.10)
	AffixTagger	0.67 (0.09)		0.78 (0.09)	0.60 (0.09)	0.73 (0.09)	0.81 (0.06)	0.66 (0.10)	0.70 (0.08)		0.81 (0.09)	0.63 (0.08)	0.61 (0.09)		0.70 (0.08)	0.52 (0.10)
	ConnectiveTagger	0.67 (0.09)		0.77 (0.09)	0.60 (0.09)	0.73 (0.08)	0.81 (0.06)	0.66 (0.10)	0.71 (0.08)		0.81 (0.09)	0.63 (0.08)	0.63 (0.09)		0.70 (0.07)	0.57 (0.10)
	MorphologicalTagger	0.68 (0.09)		0.77 (0.08)	0.60 (0.10)	0.73 (0.08)	0.81 (0.06)	0.66 (0.09)	0.71 (0.08)		0.80 (0.09)	0.63 (0.08)	0.64 (0.08)		0.71 (0.07)	0.59 (0.09)
	NegHedgeTagger	0.66 (0.09)		0.77 (0.09)	0.59 (0.10)	0.72 (0.08)	0.81 (0.06)	0.65 (0.10)	0.71 (0.08)		0.81 (0.09)	0.63 (0.08)	0.61 (0.09)		0.69 (0.08)	0.54 (0.10)
	SemanticTagger	0.68 (0.09)		0.77 (0.10)	0.61 (0.09)	0.70 (0.09)	0.78 (0.07)	0.64 (0.10)	0.72 (0.09)		0.80 (0.11)	0.65 (0.08)	0.63 (0.09)		0.69 (0.10)	0.58 (0.09)
	SyntacticTagger	0.68 (0.09)		0.78 (0.09)	0.61 (0.10)	0.72 (0.08)	0.80 (0.06)	0.65 (0.09)	0.71 (0.08)		0.80 (0.09)	0.64 (0.08)	0.63 (0.08)		0.70 (0.08)	0.58 (0.09)
	CombinedTagger	0.69 (0.09)		0.77 (0.10)	0.62 (0.09)	0.74 (0.08)	0.81 (0.07)	0.68 (0.09)	0.73 (0.08)		0.81 (0.10)	0.66 (0.07)	0.65 (0.08)		0.71 (0.08)	0.60 (0.09)

**Table 3:** The F1 score of different named entities with different features on *Comb* dataset.

Feature group	AE mean (SD)	Medication mean (SD)	Dosage mean (SD)	Frequency mean (SD)	Route mean (SD)	Duration mean (SD)	Indication mean (SD)	OSSD mean (SD)	Treatment mean (SD)	Overall mean (SD)
Default	0.70 (0.10)	0.82 (0.10)	0.59 (0.35)	0.57 (0.46)	0.36 (0.33)	0.20 (0.42)	0.57 (0.12)	0.44 (0.45)	0.60 (0.52)	0.71 (0.08)
Affix	0.69 (0.11)	0.81 (0.12)	0.58 (0.37)	0.59 (0.45)	0.55 (0.37)	0.40 (0.52)	0.57 (0.09)	0.51 (0.44)	0.60 (0.52)	0.70 (0.08)
Connective	0.70 (0.10)	0.81 (0.10)	0.69 (0.31)	0.57 (0.46)	0.44 (0.36)	0.20 (0.42)	0.60 (0.15)	0.44 (0.45)	0.60 (0.52)	0.71 (0.08)
Morphological	0.70 (0.10)	0.82 (0.10)	0.57 (0.35)	0.59 (0.45)	0.32 (0.32)	0.20 (0.42)	0.62 (0.12)	0.47 (0.43)	0.60 (0.52)	0.71 (0.08)
NegHedge	0.69 (0.10)	0.82 (0.10)	0.56 (0.36)	0.59 (0.45)	0.36 (0.33)	0.20 (0.42)	0.59 (0.11)	0.50 (0.43)	0.60 (0.52)	0.71 (0.08)
Semantic	0.71 (0.11)	0.82 (0.11)	0.56 (0.35)	0.65 (0.40)	0.34 (0.33)	0.30 (0.48)	0.64 (0.13)	0.43 (0.39)	0.60 (0.52)	0.72 (0.09)
Syntactic	0.70 (0.10)	0.81 (0.11)	0.61 (0.35)	0.59 (0.45)	0.32 (0.31)	0.34 (0.47)	0.58 (0.11)	0.44 (0.45)	0.60 (0.52)	0.71 (0.08)
All	0.72 (0.10)	0.83 (0.11)	0.61 (0.37)	0.59 (0.44)	0.32 (0.31)	0.34 (0.47)	0.65 (0.11)	0.55 (0.39)	0.60 (0.52)	0.73 (0.08)

### Annotation Disagreements

#### Overview

We manually analyzed the annotation disagreements and found they can be organized into three main categories: (1) boundary inconsistencies –disagreement due to assignment of inconsistent boundaries to entities; (2) missed named entity annotations –disagreement where one of the annotators annotated an entity and the other annotator missed it; (3) inconsistent named entity annotations –disagreement due to inconsistent categorization of entities.

There were a total of 2955 disagreed token instances, of which 1591 (53.84%) were related to *AE* and *medication* named entities.

#### Boundary Inconsistencies

We found that inconsistencies related to boundary accounted for nearly 13.94% (412/2955) of disagreement. In all the examples in the article, the named entity instance is shown in italics and the named entity type is shown within the “[]”.

In Textbox 1 Example 1, AnnLing annotated “three hour” as *duration* and “infusion” as *route*, AnnPhy annotated “three hour infusion” as *duration* only. This inconsistency exemplifies differences between the linguist and the physician. While the linguist can separate the linguistic differences between different named entities, we found that physicians (both ZFL and SB) frequently overlook the differences, which leads to inconsistent annotations.

**Examples.**

*Example 1:* She received approximately less than two minutes of therapy with intravenous Taxol (paclitaxel), 280 mg in a *three hour* [duration] *infusion* [route] for phase IIID ovarian cancer, when the symptoms occurred.

*Example 2:* The patient then became *lightheaded* [AE], *collapsed* [AE], and was *unconscious* [AE].

*Example 3:* Investigator considers that *haematologic toxicity* [AE] of methotrexate could be increased by interaction with apranax (naproxene) and sintrom (acenocoumarol).

*Example 4:* On [words marked], the patient died, presumed to be a result of *cardiogenic shock* [AE]. Prior to death, the patient was noted for having an increase in troponin T level, and found to be more unresponsive.

*Example 5:* Moderate anaphylactoid symptom appeared after administration of docetaxel and recovered later. After the end of administration, convulsion appeared. Anti-convulsion agent could not be administered due to *allergy* [AE].

*Example 6:* …days after the last Vinorelbine intake patient was hospitalized due to *NCI/CTC grade 4 neutropenia* [AE] without *fever* [OSSD]…

#### Missed Named Entity Annotations

Missed named entity annotation was the major cause for disagreement. Among 2955 disagreed token instances, 2355 or approximately 79.69% belong to this category. Table 4 shows instances of *medication* that were annotated by one annotator and missed by other. Examples 1-5 (Table 4) were annotated by AnnPhy but missed by AnnLing; examples 6-10 (Table 4) were annotated by AnnLing but missed by AnnPhy.

AnnLing explained that “blood transfusion”, “fluids”, and “red packed cells” shown in examples 1, 2, and 5 were not *medication*, but referred to a kind of treatment or medical procedure. In example 3, AnnLing missed annotating “normal saline” as *medication*. In example 4, “oxygen” was not annotated because AnnLing felt it did not represent *medication.* Annotators did not reach any consensus on annotating “oxygen” as *medication* or not. The differences here exemplify the strength of the physician as a domain-expert who may interpret the semantics of EMR notes more accurately than the linguist.

In examples 7, 8, and 10, in Table 4, AnnPhy did not annotate “treatment”, “Re-exposure”, and “chemotherapy” as these entities were anaphoric references; AnnLing, being a linguist, annotated these anaphoric references as *medication*. In example 6 (Table 4), AnnLing annotated “drug” as *medication* but AnnPhy did not annotate the entity because the text did not refer to any *medication*. Later, AnnLing agreed that where there is mention of entities but they do not refer to specific entities, such as “drug” in example 6, they should not be annotated. Example 9 in Table 4 was a special case where “concomitant drug” refers to the role or function of the drug, “Solupred”, rather than referring to a drug. AnnPhy did not annotate such instances. These examples demonstrated that annotating medical texts is a complex and cumbersome task. Further refinement of guidelines in such instances may improve the consistency of annotations.

**Table 4:** Disagreement in medication annotation (medication text is italicized).

Annotation	Medication annotation
Annotated by AnnPhy but not annotated by AnnLing	1. Given multiple *blood transfusions* (hemoglobin: 4.8). 2. Pressors continued with *fluids*. 3. He was admitted to the hospital and hydrated with *normal saline*. 4. The event was treated with steroids and *oxygen*. 5. Pancytopenia, treated with G-CSF, erythropoetin, and *red packed cells*.
Annotated by AnnLing but not annotated by AnnPhy	6. Causality assessment *drug* relationship is unable to determine for Taxol. 7. The 4th previous courses of *treatment* were well tolerated. 8. During the first infusion of paclitaxel, the patient experienced a decrease in blood pressure and was unconscious for a short while**.** *Re-exposure* elicited the same symptoms. 9. The *concomitant drug* prescribed was oral Solupred instead of Solumedrol. 10. A female patient possibly received non-therapeutic dosages of intravenous Taxol (paclitaxel), Paraplatin (carboplatin), and/or Platinol (cisplatin) for the treatment of ovarian cancer and subsequently expired. It was reported that the pharmacist possibly diluted *the chemotherapy* improperly.

### Inconsistent Named Entity Category Annotations

We have annotated a total of nine categories of named entities, as shown in Table 1. The third type of inconsistency was caused by inconsistent named entity assignments. Among 2955 disagreed token instances, 188 (6.36%) belong to inconsistent named entity categorization. We manually examined few instances and Examples 2-6 in Textbox 1 show the annotated sentences where inconsistency occurred. Example 2 in Textbox 1 is an example where both annotators agreed on the AE annotation.

Example 3 in Textbox 1, however, shows an instance where AnnPhy and the tiebreaker agreed on “haematologic toxicity” as an AE whereas AnnLing did not initially annotate the entity. This inconsistency suggests that domain knowledge is required for annotation. After discussion with two other annotators, AnnLing agreed that “haematologic toxicity” should be annotated an AE.

Example 4 in Textbox 1 shows an instance where AnnLing and the tiebreaker agreed on “cardiogenic shock” as an AE but AnnPhy annotated it as OSSD. AnnPhy argued that “cardiogenic shock” caused “death” and therefore “death” should be an AE and “cardiogenic shock” is the reason for death and therefore was annotated as OSSD. This example shows the complexity of clinical cause.

In Textbox 1 Example 5, the tiebreaker annotated “allergy” as an AE, whereas AnnPhy annotated it as OSSD and AnnLing did not annotate it as an AE because it refers to the patient’s history of “allergy” and does not represent a current instance of AE. We will need to refine our annotation guideline to add current or past status in addition to the named entity annotation.

Example 6 in Textbox 1 shows an instance of boundary inconsistency. AnnPhy and AnnLing both annotated “NCI/CTC grade 4 neutropenia without fever” as an AE whereas the tiebreaker annotated “NCI/CTC grade 4 neutropenia” as an AE and “fever” as OSSD. This is a case in which annotators interpret clinical texts differently. Such an inconsistency is difficult to address due to the nature of ambiguity in clinical texts.

### Error Analyses

**Figure 3:**
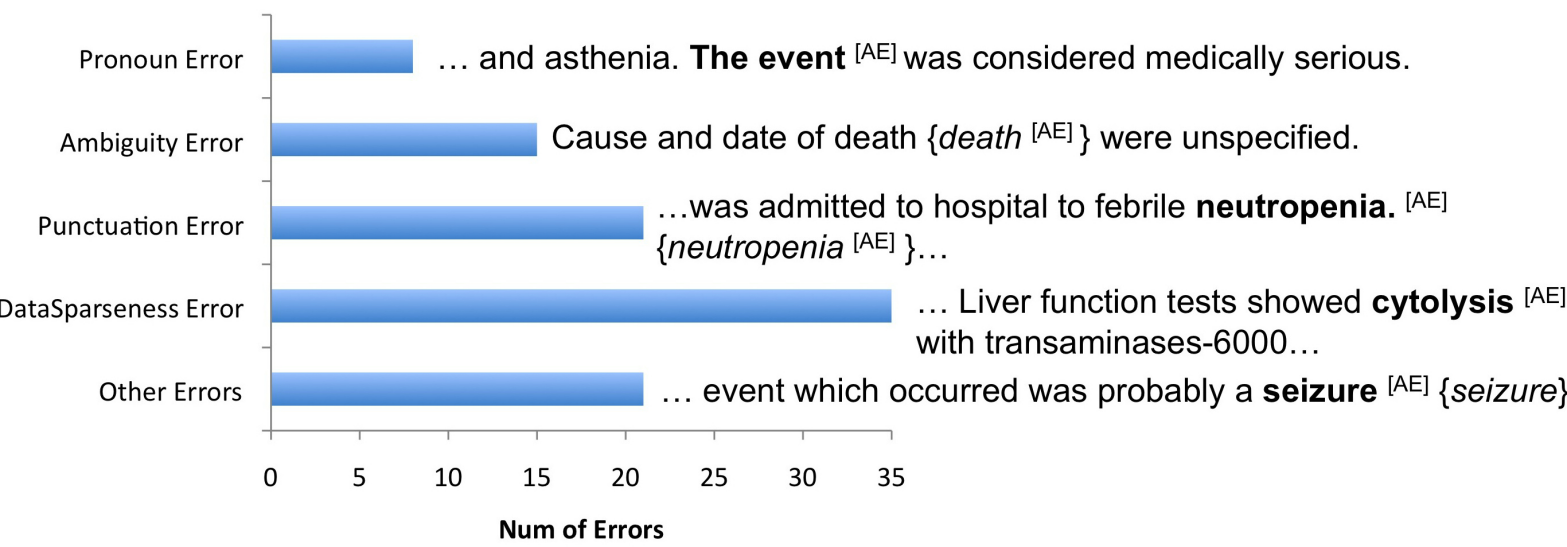
Error categories, their frequency, and an illustrative example of error category on 100 randomly sampled instances. The annotated entities are shown in bold, the annotated named entity type is shown within “[]” and tagger output is {italicized}. AE: adverse events.

For error analyses, we focused on *CombinedTagger* because it yielded the highest performance (as shown in Table 2) and the *Comb* dataset because it contained annotations agreed on by both annotators. We randomly selected 100 named entities predicted wrongly by *CombinedTagger* and manually analyzed them. As shown in Figure 3, we group the named entities into a total of five types of errors and give an illustrative example for each. In all examples, annotated named entities are shown in bold, the tagger output in {*italicized*} and the named entity type is shown within “[]”. The leading type of error was data sparseness (35%). Data sparseness is a common problem and the major cause of poor performance. For instance, the gold standard consisted of a number of singleton instances (instances that appear only once) like “cytolysis”, “sodium chloride solution 0.9% 100ml”, and “neoplasm of unspecified nature of respiratory system” that created sparseness in the data.

The second cause of error was inconsistent inclusion of punctuation (21%). The gold standard had inconsistency in inclusion of punctuation (eg, a period [.] in “neutropenia.”) as a part of a named entity. This boundary inconsistency reduced the overall performance. Figure 3 shows an instance where the gold standard included a period as part of named entity “neutropenia.” but the tagger failed to include it (“neutropenia”). This was followed by an error caused by ambiguous named entities (15%). The instances in the gold standard that were assigned to multiple named entity categories resulted in ambiguous entities. For example, “death” was annotated as either *AE* or *OSSD.* This could have confused the ML algorithm and yielded a lower performance. In Figure 3, the instance “death” was not annotated as *AE* in the *Comb* dataset due to disagreements between annotators, but the tagger identified it as an *AE*. The missed pronoun annotations such as “the event” contributed to 8% of the errors. The final category was other type of errors (21%), for which the exact cause of error could not be determined. In Figure 3, “seizure” was annotated as an *AE* but the tagger failed to identify it. The exact cause for miscategorization could not be determined.

### Annotation Inconsistencies

As predicted, annotation inconsistency played an important role on AETaggers’ performance as our Pearson correlation results (coefficient of 0.73) show that the IAA value (Cohens’ kappa) is positively correlated with the machine learning performance of named entity recognition. This is not surprising because inconsistent annotations confuse the machine learning systems.

Our manual analysis of inconsistency revealed that nearly 20% of errors were due to inconsistent inclusion of punctuation in annotation. When we removed the inconsistency in punctuation, the F1 score of *CombinedTagger* increased from 0.73 to 0.79, which was statistically significant (*t* test, *P*=.001). Although the missed pronoun annotations of *AE* and *medication* can be fixed readily, they also contributed to the lower performance of the tagger.

### Data Sparseness

Data sparseness is a common problem and the major cause of poor performance. The performance of AETagger was positively correlated with the size of the annotated data for each named entity (a Pearson correlation coefficient of 0.64). In the cases of *frequency*, *duration, OSSD,* and *treatment* entities, data was very sparse (Table 1) and taggers showed low performance on these named entities. In addition to low performance, data sparseness also contributed to a higher standard deviation (Table 3). When the training data incorporate instances of a named entity but the testing data do not, the precision decreases. When the training data misses instances of a named entity but the testing data do not, then recall suffers.

### Learning Features

To further understand the contribution of learning features on the performance of AETagger, we first trained the tagger with all the features and used it as a baseline system (*CombinedTagger*). We then removed each feature category one at a time. Table 5 shows the performance of taggers with each feature category removed. Consistent with Table 3, the results show that each feature contributed to the performance differently.

**Table 5:** The precision, recall, and F1 score of Taggers with feature categories removed one at a time on each of the four annotated data sets.

Tagger	AnnPhy Mean (SD)			AnnLing Mean (SD)			Combined Mean (SD)			Tie Mean (SD)		
	F1	Precision	Recall	F1	Precision	Recall	F1	Precision	Recall	F1	Precision	Recall
All features	0.67 (0.09)	0.77 (0.10)	0.62 (0.09)	0.74 (0.08)	0.81 (0.07)	0.68 (0.09)	0.73 (0.08)	0.81 (0.10)	0.66 (0.07)	0.65 (0.08)	0.71 (0.08)	0.60 (0.09)
No affix features	0.68 (0.09)	0.76 (0.10)	0.62 (0.09)	0.71 (0.10)	0.78 (0.07)	0.65 (0.11)	0.71 (0.09)	0.79 (0.11)	0.64 (0.09)	0.64 (0.08)	0.70 (0.08)	0.60 (0.08)
No connective features	0.69 (0.09)	0.77 (0.10)	0.62 (0.09)	0.74 (0.08)	0.81 (0.06)	0.69 (0.09)	0.73 (0.08)	0.81 (0.10)	0.66 (0.07)	0.65 (0.08)	0.71 (0.08)	0.60 (0.09)
No morphological features	0.69 (0.09)	0.78 (0.10)	0.62 (0.09)	0.73 (0.08)	0.81 (0.06)	0.66 (0.09)	0.73 (0.08)	0.82 (0.10)	0.66 (0.07)	0.65 (0.08)	0.72 (0.08)	0.60 (0.08)
No negation and hedge features	0.68 (0.09)	0.77 (0.10)	0.62 (0.09)	0.74 (0.08)	0.81 (0.07)	0.68 (0.09)	0.72 (0.08)	0.81 (0.10)	0.65 (0.07)	0.64 (0.09)	0.71 (0.09)	0.59 (0.09)
No semantic features	0.67 (0.08)	0.77 (0.08)	0.60 (0.09)	0.74 (0.08)	0.82 (0.05)	0.68 (0.10)	0.71 (0.08)	0.80 (0.09)	0.64 (0.08)	0.64 (0.08)	0.71 (0.07)	0.59 (0.08)
No syntactical features	0.68 (0.09)	0.77 (0.10)	0.61 (0.09)	0.73 (0.08)	0.80 (0.07)	0.68 (0.09)	0.71 (0.09)	0.80 (0.11)	0.64 (0.08)	0.64 (0.08)	0.70 (0.09)	0.58 (0.09)

## Discussion

### Principal Findings

Our results show that medication and adverse events can be reliably annotated (Cohen’s kappa value of .64-.95 IAA as shown in Table 1) in the FAERS narratives. Many named entities (eg, *indication*) that had shown low annotation agreements in the i2b2 challenge [63] had good annotation agreements in our dataset. The improvements were attributed to improved annotation guidelines and the quality and domain specificities of the FAERS narratives.

With a good IAA, we still found room to further improve the annotation guideline. For example, our error analyses (Figure 3) show that inconsistencies were introduced by annotation boundary; therefore it can be further refined. Although *medication* had the highest IAA (.95), our analysis (Table 4) found that the inconsistency in *medication* was introduced by whether instances like “fluids” could be considered as medication or not. In the future, we may separate *medication* into two classes: *strict medication* and *unstrict medication*. The names and mentions of all drugs appearing in the United States Pharmacopeia will belong to *strict medication;* any substances or chemicals—including oxygen, fluids, drinks, and others–given to patients during the treatment will be classified as *unstrict medication.* Refining the guideline to annotate previous and potential AEs like “allergy” (Example 5) may further reduce the inconsistency.

We explored various ML methods and compared them with a baseline string matching and Metamap-based systems to assess the complexity of the task. The CRF-based tagger had the best performance. Further analyses of the CRF tagger found that data sparseness affected the taggers’ performance (Figure 3). For example, the standard deviation of *treatment* is high because we found that the testing data did not incorporate *treatment* instances. Similar behavior was also observed for other sparse entities (Table 3).

Using the best performing ML technique, we explored a variety of features (Table 2 and Table 3). The features had a mixed effect on the performance of the taggers and the combination of all the features improved overall performance slightly. This suggests the robustness of the default features for CRFs. Since most of the features were extracted automatically (eg, negation, hedge cues, and discourse connectives were extracted using the taggers [77,78] and parser [79] we developed), the accuracy of the extracted features played an important role in overall performance of the tagger. To avoid the noise introduced by automatic feature extraction, one may explore the features manually annotated such as POS in the PennTree Bank [80]. This is, however, expensive. An alternative is to further improve the performance of the BioNLP systems for feature extraction.

Throughout the study, we found that additional features may be further included. For example, we observed that *OSSD* most often appeared in the patient’s medical history. We therefore added a feature representing patient history and found that the performance of the *CombinedTagger* on *OSSD* increased 1.2% (results not reported in the Result section), although the increase was not statistically significant (*t* test, *P*=.25).

### Limitations

Our study has limitations. The AETaggers were trained on the FAERS corpus we constructed. Like any other NLP system, the performance of the tagger on other types of EMRs can vary based on the structure and content of the narrative text. On the other hand, since our selection of the FAERS corpus was through a random process, we speculate that the data is representative. Although the taggers performed well, the training and evaluation was based on a relatively small training dataset. In the future, we would increase the size of the training data and explore other semi-supervised machine learning approaches to further improve the performance.

### Conclusions

In this study, we developed an annotation guideline for medication and adverse event information from the FAERS narratives, our annotation of 122 FAERS narratives (a total of approximately 23,000 tokens) showed a reliable inter-rater annotation agreement (an overall kappa of .82). We then developed machine learning models for automatically extracting medication and adverse event information from the FAERS narratives. We explored utilizing different learning features in the machine learning models. The results showed that features such as syntactic, semantic, morphological, and affix improved the performance and the best performing system had an overall F1 score of 0.73. In the future, we would like to refine further the annotation guideline, explore additional features and increase the annotation size to improve system performance. We will also explore approaches for normalizing the entities by mapping them to standard terminologies like MedDRA and identify the causal relation between a medication and an adverse event.

## Multimedia Appendix 1

Annotation Guideline.

## Abbreviations

ABNER: A Biomedical Named Entity Recognizer
ADE: adverse drug event
AE: adverse event
CRF: Conditional Random Fields
EMR: Electronic Medical Records
FAERS: FDA Adverse Event Reporting System
FDA: Food and Drug Administration
IAA: inter-annotator agreement
MedDRA: Medical Dictionary for Regulatory Activities
ML: machine learning
NB: Naïve Bayes
NLP: natural language processing
OSSD: Other Signs, Symptoms and Diseases
POS: part of speech
SVM: Support Vector Machines
