# Supplementary material for: Automatically Recognizing Medication and Adverse Event Information From Food and Drug Administration’s Adverse Event Reporting System Narratives
Source: JMIR Med Inform. 2014 Jun 27;2(1):e10. doi: 10.2196/medinform.3022 (PMC4288072; doi:10.2196/medinform.3022)
Supplement: Supplementary file 1 [file medinform_v2i1e10_app1.pdf]

Extract Adverse Drug Events from FDA Adverse Event Reporting System Narratives

Annotation Guidelines

1. Overview

The aim of this project is to build an Intelligent Natural Language Processing System for Pharmacovigilance. The hypothesis underlining is FDA AERS incorporate adverse drug events (ADEs) that can be reliably recognized by domain experts and we will annotate such narratives regarding medication exposure, AEs and ADEs. We will in addition map AEs to the external knowledge resources CTCAE 4.0 and the MedDRA. We will also annotate linguistic features including discourse relations.

The i2b2 Medication Challenge Annotation Guideline is used as baseline guideline. You can access that at Lancet Google project (http://lancet.googlecode.com/files/Preliminary.Annotation.Guidelines.7.9.pdf). Please make sure that you have read it before annotation.

Generally, we follow that guideline to annotate drug name and medication fields. There are some exceptions from i2b2 guideline are summarized in the section named Annotation Practices.

2. Annotation Practices

The signs, symptoms or disorders are annotated and classified into 4 classes: adverse effects, beneficial effects, indications and other terms (Leaman et al. 2010). Adverse effects could be any expression of signs, symptoms or disorders, including pronouns that refer to any of the above. For each adverse drug reaction report, there must be one adverse effect. When there are hints to indicate the relationship between certain drug and adverse effect, the two entities are link. If not, just let it alone.

When the adverse effects and drug appeared in same sentence, generally it would be annotated. For example,

```
During the first infusion of paclitaxel (12 Jun 2003), the patient experienced a decrease in blood pressure and was unconscious for a short while. (T130)
```

However, when there is clear negative declaration, the relationship would not be annotated. For example,

```
BMS Medical Monitor causality: Death not-likely related to Taxol and carboplatin therapies.
```

For the drugs and adverse drug reactions that appear in two separate sentences, the relationship would not be annotated even there is clear clue for that. For example,

```
The event myocardial infarction was changed to haemorrhage of
```

```
digestive tract. Intravenous dexamethasone sodium phosphate was
considered a suspect drug (dosage and therapy dates not provided).
```

The adverse effects are mapped to the concepts from two lexicons: CTCAE 4.0 (Trotti et al. 2003) and MedDRA (Mozzicato 2009). When pronouns are annotated, the adverse effects are mapped to 'Adverse event' with a MedDRA code 10060933.

For medication name and related medication fields' annotation, generally, i2b2 2009 annotation guideline is followed (Uzuner et al. 2009). For example, the annotated medications must be experienced by the patient and the drug names include brand name, generics, ingredients or collective name. The difference is that we would annotate the allergic drugs because allergy is part of adverse events. For the medication fields, we do not follow the "two lines window" rules. In addition, the reason for suspected drugs was specialized as indication. Adverse effects may or may not be reasons for drugs.

In the context or section talking about the causality of the adverse event, the relationship between adverse side effects and suspected drugs is built and an outcome value for each annotated adverse effect would be assigned based on the context. There are three choices for the slot: recover, not completely recover and die.

3. Software checklist

3-1 Installation of Protégé and Knowtator *

Software	URL	Document
**Protégé**	http://protege.cim3.net/download/old-releases/3.3.1/basic/	
**Knowtator**	http://knowtator.sourceforge.net/	http://knowtator.sourceforge.net/install.shtml
**MedDRA browser**	http://www.meddramsso.com/subscriber_download_tools_browser.asp	Need MedDRA131E, import the folder named MedAscii.

		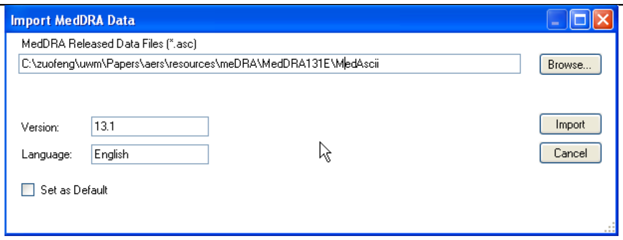
**I2b2 medication annotation guideline**	http://lancet.googlecode.com/files/Preliminary.Annotation.Guidelines.7.9.pdf	

* The Windows version of the software have been downloaded and saved in **resource** folder.

References

Leaman, R. et al., 2010. Towards Internet-Age Pharmacovigilance: Extracting Adverse Drug Reactions from User Posts in Health-Related Social Networks. In *Proceedings of the 2010 Workshop on Biomedical Natural Language Processing*. Uppsala, Sweden: Association for Computational Linguistics, pp. 117–125. Available at: http://www.aclweb.org/anthology/W10-1915.

Mozzicato, P.[., 2009. MedDRA: An Overview of the Medical Dictionary for Regulatory Activities. *Pharmaceutical Medicine*, 23, 65-75.

Trotti, A. et al., 2003. CTCAE v3.0: development of a comprehensive grading system for the adverse effects of cancer treatment. *Seminars in Radiation Oncology*, 13(3), 176-181.

Uzuner, O., Solti, I. & Xia, F., 2009. i2b2 medication extraction challenge preliminary annotation guideline. Available at: http://lancet.googlecode.com/files/Preliminary.Annotation.Guidelines.7.9.pdf [Accessed July 3, 2013].
